# Scalable and distributed strategies for socially distanced human mobility

**DOI:** 10.1007/s41109-021-00437-9

**Published:** 2021-12-13

**Authors:** Satyaki Roy, Preetam Ghosh

**Affiliations:** 1 University of North Carolina, Chapel Hill, USA; 2 Virginia Commonwealth University, Richmond, USA

**Keywords:** Social distancing, Network science, Clustering, Sampling, Parallelization

## Abstract

COVID-19 is a global health crisis that has caused ripples in every aspect of human life. Amid widespread vaccinations testing, manufacture and distribution efforts, nations still rely on human mobility restrictions to mitigate infection and death tolls. New waves of infection in many nations, indecisiveness on the efficacy of existing vaccinations, and emerging strains of the virus call for intelligent mobility policies that utilize contact pattern and epidemiological data to check contagion. Our earlier work leveraged network science principles to design social distancing optimization approaches that show promise in slowing infection spread however, they prove to be computationally prohibitive and require complete knowledge of the social network. In this work, we present scalable and distributed versions of the optimization approaches based on Markov Chain Monte Carlo Gibbs sampling and grid-based spatial parallelization that tackle both the challenges faced by the optimization strategies. We perform extensive simulation experiments to show the ability of the proposed strategies to meet necessary network science measures and yield performance comparable to the optimal counterpart, while exhibiting significant speed-up. We study the scalability of the proposed strategies as well as their performance in realistic scenarios when a fraction of the population temporarily flouts the location recommendations.

## Introduction

Severe Acute Respiratory Syndrome Coronavirus 2, stated as a global pandemic by the World Health Organization, has infected over 200 million people and led to nearly 4.5 million deaths worldwide. As lockdown and social distancing techniques (https://www.who.int/news/item/13-10-2020-impact-of-covid-19-on-people’s-livelihoods-their-health-and-our-food-systems) became the primary means to combat the soaring infection counts, the impending economic challenges (Overberg et al. 2020) and recent successes in fast-track vaccine development (https://news.harvard.edu/gazette/story/2020/12/anthony-fauci-offers-a-timeline-for-ending-covid-19-pandemic/) has encouraged the world leaders to lift lockdown restrictions. However, the late wave of infection surge in several countries, lingering doubts over the effectiveness and health impacts of the vaccines and new virus strains necessitate intelligent public mobility policies that harness contact patterns and epidemiological information to check the impending threats of contagion during present and future outbreaks (https://www.healthline.com/health-news/experts-concerned-a-4th-covid-19-wave-may-be-building).

The lack of foresight and preparedness on the part of the world leaders resulted in the absence of coordinated action plans or public policies. While the world was relying on the findings from the geneticists, doctors and health officials to design makeshift regulations, the epidemiologists, statisticians, and computer scientists explored the socioeconomic and demographic factors contributing to this rapid spread (Adhikari et al. 2020). These efforts included computational and machine learning techniques to predict trends on spread dynamics from epidemiological and clinical data (Wynants 2020; Holmdahl and Buckee 2020; Alimadadi et al. 2020; Randhawa et al. 2020). Their findings lent insights into the epidemiology, causes, clinical manifestation and control measures and helped identify vulnerable communities. Regression analysis and computational approaches were employed as means to gauge effects of testing (Khan et al. 2020; Roy et al. 2021a) and lockdown (Roy and Ghosh 2020) on the pandemic, while unsupervised machine learning and natural language processing approaches broadened our understanding of disease transmissibility and economic challenges (Wang et al. 2020; Roy et al. 2021b).

The accuracy of the parameters of the epidemic models as well as the latter’s capability in modeling the epidemiological trends have been key areas of investigation. Holmdahl discussed the constant effort on the part of scientists to refine methods to learn spread dynamics of infectious diseases (Holmdahl and Buckee 2020). Clearly, the predictions from the epidemic models are contingent on factors such as knowledge of demography, infectivity of the virus, accuracy of testing, etc. For instance, Bedi et al. modified the Susceptible-Exposed- Infected-Recovered (SEIR) epidemic model by assuming exposed individuals to be infective and compared the accuracy of their model against that of a Long Short-Term Memory (LSTM) model (Bedi et al. 2020). Gharakhanlou et al. investigated the spread dynamics in Iran by employing agent-based simulation and recommended mitigation measures (Gharakhanlou and Hooshangi 2020), while Ghanam et al. studied the role of government intervention (Ghanam et al. 2020). Furthermore, efforts have been made to analyze the interrelationship between vaccinations, lockdown, mobility and spread (Roy et al. 2021c; Lattanzio and Palumbo 2020) and curb spread through contact-tracing based mobile applications (Kretzschmar et al. 2020; Ferretti et al. 2020; Ahmed et al. 2020; Vax 2014; Nadini et al. 2020; Koppeschaar and Colizza 2017; Dalton et al. 2006). These applications rely on the duration of contact, proximity between individuals and online surveys recording location, patient health and demographic details to identify risk factors. Earlier, we proposed three optimization approaches on social networks that apply network science principles to mitigate contagion by guiding human mobility (Roy et al. 2021d). We carried out simulation experiments using realistic human mobility models and the New York City map to demonstrate that the approaches effectively slow contagion spread. We also designed a mobile application, *MyCovid* (Roy et al. 2021d; Roy 2021) that is presently being deployed to validate the system performance in a realistic setting. However, these approaches face scalability and centralization challenges for large populations.

### Contributions

In this work, we present social distancing strategies that optimize the location of individuals residing in urban spaces, such as grocery stores, bus queues, auditoriums, etc., where individuals are likely to engage in social contacts leading to contagion. Our initial efforts in this direction (Roy et al. 2021d) demonstrated three proposed social distancing approaches leveraging network science principles such as *homophily*, *network clustering*, etc. These approaches minimize the number of social ties between the vulnerable (i.e., susceptible individuals) and the vectors of infection (i.e., infected individuals), thereby dampening the rate of infection spread. However, this work is challenged on two fronts: (1) the number of parameters in the optimization scale linearly with the number of individuals, making them computationally prohibitive for large populations and (2) they rely on the knowledge of the entire social network topology. We address these issues in the scalable and distributed social distancing strategies that leverage Markov Chain Monte Carlo Gibbs sampling and grid-based spatial parallelization.

We carry out simulation experiments to show the efficacy of the proposed strategies. We analyze how the system parameters, namely, convergence index and number of grids, can be utilized to tune the optimality vs. scalability trade-off. We gauge the performance of the distributed and sampling strategies in terms of the running time in seconds, optimization score (defined in terms of potential contact between susceptible and infected individuals) as well as the rate of contagion over time in terms of cumulative population of infected, recovered, and dead individuals as per the Susceptible-Exposed-Infected-Recovered-Dead (SEIRD) epidemic model (discussed in “SEIRD Epidemic Model” section). Finally, we show the scalability of the approaches and the effect of a fraction of individuals flouting the recommendations of the system on contagion.

This paper is organized as follows. In “Preliminary Concepts and System Model” section, we discuss the SEIRD epidemic model, preliminary concepts of network science and the system model. In “Approach” section, we present the three social distancing optimization approaches, followed by the scalable and distributed solutions. Sections 4 and 5 deal with the experimental results and discussions. We draw the conclusions in Sect. 6.

## Preliminary concepts and system model

Let us first discuss the SEIRD epidemic model, network science concepts (namely, network clustering and homophily) and the system model.

### SEIRD epidemic model

The SEIRD model can represent the evolution of the susceptible (S), exposed (E), infected (I), recovered (R) and dead (D) populations (Hethcote 2000). The individuals in S transition to E with rate *β*, while E transition to I with probability *σ*; I transition to R with probability *γ* × (1 − *α*) and D with probability γα. In other words, *γ* denotes the proportion of infected that transition to other states, and *α* is the fraction of those individuals to die. We show the equations corresponding to the state transitions, where *R*_0_ is the basic reproduction number ranging between 3 and 6 and *β* = *γ* × *R*_0_ (Korolev 2021; Early release-high contagiousness and rapid spread of severe acute respiratory syndrome coronavirus 2020).

1 $$S\to E$$

2 $$E\to I$$

3 $$I\to R$$

4 $$I\to D$$

Arroyo-Marioli et al. presented an approach to track the rate of contagion in terms of *effective reproduction number* and *growth rate* (defined as rate of increase in daily infection numbers over time) (Arroyo-Marioli et al. 2021). They represented the new infection count at time *t* in a population of *N,* as

5 $${I}_{t}\to {I}_{t-1}+\frac{{\beta }_{t}\times {I}_{t-1}\times {S}_{t-1}}{N}-\gamma {I}_{t-1}$$

6 $${\text{Hence}},I_{t} - I_{t - 1} \to \frac{{\beta_{t} \times I_{t - 1} \times S_{t - 1} }}{N} - \gamma I_{t - 1}$$

Given basic reproduction number ${R}_{0} = \beta$ and growth rate ${G}_{t}=\frac{{I}_{t}-{I}_{t-1}}{{I}_{t}}$, the effective reproduction number at time $t ({R}_{t})$ is calculated as:

7 $${R}_{t}={R}_{0}\times \frac{{S}_{t-1}}{N}= \frac{{\beta }_{t}}{\gamma } \times \frac{{S}_{t-1}}{N}$$

Plugging *R*_*t*_ in Eq. 6, we get,

8 $${G}_{t}=\frac{{I}_{t}-{I}_{t-1}}{{I}_{t-1}}=\gamma \times {R}_{t}-\gamma$$

9 $${R}_{t}=\frac{{I}_{t}-{I}_{t-1}}{{I}_{t-1}}=1+\frac{1}{\gamma }\times {G}_{t}$$

### Key network science concepts

Given an undirected *G* (*V, E*), we discuss the network science concepts that are incorporated by the proposed mobility optimization approaches (details discussed in “Social distancing optimization” section).

#### Network clustering

It is the tendency of nodes to form dense communities within *G*, measured in terms of the number of triads they participate in Holland and Leinhardt (1971), as follows:

10 $$\begin{aligned} & \zeta \left( {G,u} \right) = 0 \hspace{36mm} if \hspace{2mm} \delta \left( u \right) < 2 \\ & \quad \hspace{8.5mm}=\frac{2 \times t\left( u \right)}{{\delta \left( u \right) \times \left( {\delta \left( u \right) - 1} \right)}}\quad {\text{otherwise}} \\ \end{aligned}$$

**Fig. 1:**
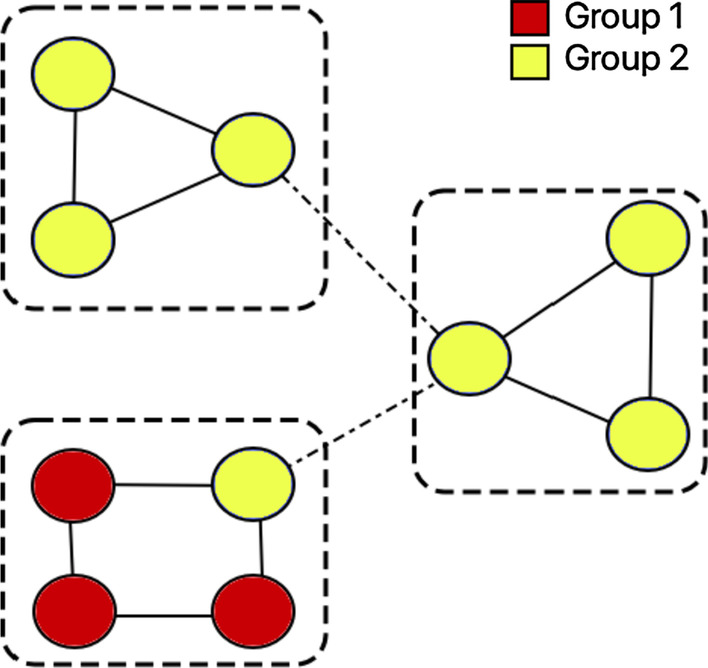
Network clustering and homophily: Three clusters demarcated by boxes; a node belongs to either group 1 or 2 (denoted by red and yellow colors, respectively.) The inter and intra-group social ties are denoted by dashed and solid lines, respectively

In this equation, *t*(*u*) and *δ*(*u*) are the number of triads participated by node *u* and degree of *u* ∈ *V*, respectively. The overall node clustering coefficient of the network (i.e., average clustering of all nodes) shown in Fig. 1, on a scale of 0 and 1, is 0*.*6.

#### Homophily

It is the tendency of nodes choosing to attach with nodes of its own *group*, defined as nodes with similar characteristics (Kim and Altmann 2017; McPherson et al. 2001; Kossinets and Watts 2009). We measure homophily in terms of *E-I index*, defined as the difference between proportion of ties between members from different groups and members from the same group (Bojanowski and Corten 2014). An E-I score of − 1 means complete homophily, while E-I score of 1 denotes complete heterophily. The network in Fig. 1 has E-I index =  − 0*.*6, making it highly homophilic.

### System model

We consider an urban space of dimension *X* × *Y* square feet, where *ν* mobile individuals are placed. We define *contact threshold d* as the maximum distance between the susceptible and infected individuals such that the susceptible individual may be exposed to the pathogen. We create social network *G*_*t*_(*V, ϵ*_*t*_), where *V* is the set of *ν* nodes (each representing an individual) and *ϵ*_*t*_ is the set of temporal edges, where an edge (*u, v*) ∈ *ϵ*_*t*_ if individuals *u* and *v* are within threshold *d* at time *t* ∈ (experiment duration) *T*. A node, belonging to exactly one epidemic class (S, E, I, R or D) can move within a distance threshold *τ* of its current location between time *t* and *t* + 1.

## Approach

**Fig. 2:**
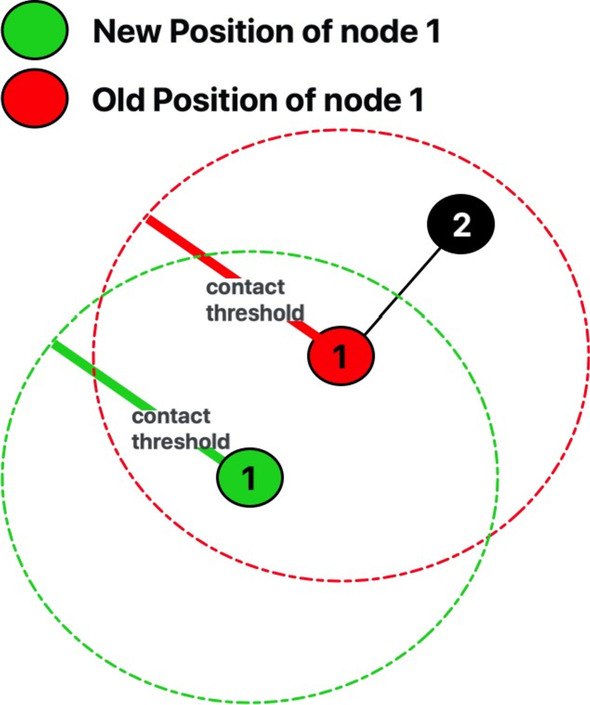
Network clustering and homophily: Three clusters demarcated by boxes; a node belongs to either group 1 or 2 (denoted by red and yellow colors, respectively.) The inter- and intra-group social ties are denoted by dashed and solid lines, respectively

Each individual carries a smart mobile device capable of communicating with other devices in the vicinity via Wi-Fi or Bluetooth. The neighbor-list of a node *u*, *n*_*t*_ (*u*), is the set of individuals that are within distance *d* at time *t*. Each individual *u* must belong to exactly one of *S, E, I,* R*, D* states, where *S* ∪ *E* ∪ *I* ∪ R ∪ *D* = *V*. (*S*, *E*, *I*, R and *D* occasionally have a subscript to denote the number of individuals in that epidemic group at the *t*-th time epoch.) Fig. 2 shows node 1 relocates to a new location (colored green) within a threshold distance (*τ* feet) of its old location (colored red).

### Social distancing optimization

The optimization approaches discussed below were originally proposed in Roy et al. (2021d). Given any social network *G*_*t*_, these approaches utilize network science principles of network clustering and homophily (see “Key Network Science Concepts” section for details) to generate new locations for individuals (and resultant network *G*_*t*+1_). The goal is to minimize the contact (i.e., links in the social network) between the susceptible and infected individuals and slow the overall contagion.

#### Direct contact approach

**Fig. 3:**
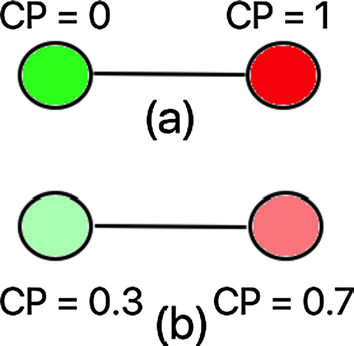
Contagion potential (CP) of connected nodes: **a** Direct contact optimization approach eliminates ties between likely susceptible (CP = 0) and infected (CP = 1) individuals; **b** Contagion potential optimization approach minimizes ties if the difference of CPs of two connected nodes is high

It eliminates the contact between the susceptible and infected individuals (see Fig. 3a and Expression 11). This is based on the premise that the infected individuals are the primary sources of contagion, and the susceptible nodes are the target.

#### Clustering approach

**Fig. 4:**
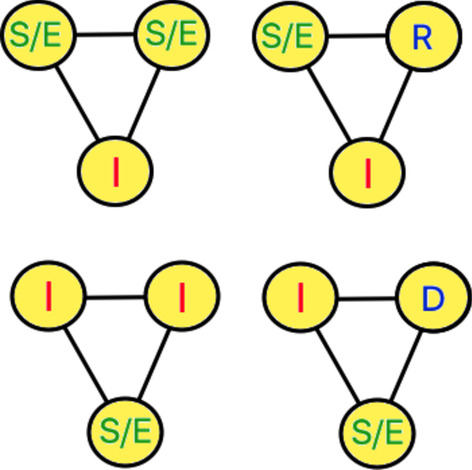
4 triangular configurations with at least one susceptible (S) (or exposed (E)) and one infected individual (I)

This approach eliminates clusters containing infected individual(s) from the social networks, by re-positioning of nodes (see Expression 12). Recall from “Key Network Science Concepts” section clustering is quantified by the triangle participation of the nodes. Figure 4 shows the four-triangle configurations eliminated by the optimization through node re-positioning.

#### Contagion potential approach

It takes into account the scenario (similar to the model presented in Bedi et al. (2020)) where a person may act as spreader without being tested and identified as infected. We define *contagion potential* (CP) of node *u* (on a scale of 0 and 1) as its likelihood of acting as spreader. Instantaneous CP is calculated in terms of the number of contacts with individuals with high CP, as follows:

$$\begin{aligned} & \hspace{10mm} = 0 \quad \hspace{28mm} if \hspace{5mm} t = 0 \\ & P_{t} \left( u \right) = 1 \quad \hspace{28mm} if \hspace{5mm} t \ge 1, u \in I \\ & \hspace{10.5mm} = \frac{{ \mathop \sum \nolimits_{{v \in n_{t} \left( u \right)}} P_{t - 1} \left( v \right)}}{{M_{t} }}\quad {\text{otherwise}} \\ \end{aligned}$$

*M*_*t*_ is the maximum number of neighbors of any node at time *t*. Overall CP till time *T, Z*_*T*_ is estimated as the mean over the instantaneous values, as follows:

$$\begin{aligned} & \hspace{15.5mm} 0\quad \hspace{18.5mm} if \hspace{5mm} u \in R, D \\ & Z_{t} \left( u \right) = 1 \quad \hspace{18.5mm} if \hspace{5mm} u \in I \\ & \hspace{14mm}\frac{1}{T}\sum P_{t} \left( u \right)\quad {\text{Otherwise}} \\ \end{aligned}$$

**Fig. 5:**
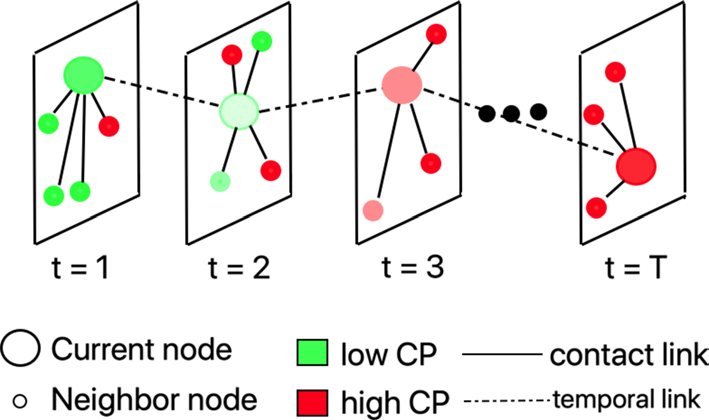
Evolution of contagion potential (CP) over time. Every panel shows the location of nodes at time t (1 ≤ t ≤ T) and the spectrum of colors—dark green, light green, light red and dark red—represent increasing CP of the observed node (large circle) on contact with infected individuals (small circles)

Figure 5 shows the evolution of the epidemic state of a node over time $t=1, 2, \dots , T$. The node (depicted as a large circle) has potential of being a spreader due to its lack of contact with other infected individuals. Consequently, it has a low CP (colored green). This approach (formulated in Expression 13) considers the fact that untested individuals may be infected, and testing can be erroneous. It employs the principle of homophily (refer “Key Network Science Concepts” section) to group nodes with similar CP into clusters and minimizes the contact between individuals with a high variation in CP. This approach is a generalization of opt-1; instead of representing the infectivity of an individual as a binary case, it assumes a continuous CP value between 0 and 1. In the experimental results discussed in Sect. 4.1, we show that the contagion potential (CP) optimization approach creates more homophilic social networks, by creating links among individuals with similar CP and eliminating links between nodes with dissimilar CPs (see Fig. 3b). This similarity of CP among clustered nodes measured in terms of E-I index minimizes the risk of contact between a potential susceptible and infected individual.

#### Optimization formulations

In Expression 11, *f* (*u, v, G*_*t*_) = 1 if nodes *u* and *v* are connected (i.e., (*u, v*) ∈ *ϵ*_*t*_) in social network *G*_*t*_, and 0 otherwise. Note that *v* ∈ *I*_*t*_, while *u* ∈ *S*_*t*_ or *E*_*t*_ because the susceptible and exposed are both asymptomatic and indistinguishable in the real world. Function *δ* (*u, v, w, G*_*t*_) in Expression 12 is equal to 1, if *u, v, w* ∈ *V* form a triangle with at least one infected node, i.e.,

1. (*u, v*)*,* (*v, w*)*,* (*u, w*) ∈ *ϵ*_*t*_, and

2. *u* ∈ *S*_*t*_*/E*_*t*_||*v* ∈ *S*_*t*_*/E*_*t*_||*w* ∈ *S*_*t*_*/E*_*t*_* and u* ∈ *I*_*t*_||*v* ∈ *I*_*t*_||*w* ∈ *I*_*t*_

The function *δ* (*u, v, w, G*_*t*_) = 0 otherwise.

11 $$\underset{{C}_{t+1}}{\mathrm{min}} \sum \sum f\left(u, v, {G}_{t}\right)$$

12 $$\underset{{\mathrm{C}}_{\mathrm{t}+1}}{\mathrm{min}} {\sum }_{u\in S,E,I }{\sum }_{u\in S. E. I;v>u}{\sum }_{u\in S. E,I, w>v}\delta (u, v, w, {G}_{t})$$

13 $$\mathrm{min} {\sum }_{\left(u, v\right)\in {E}_{t};u, v\in S.E.I}|{Z}_{t}\left(u\right)-{Z}_{t}(v)|$$

14 $$s.t. abs\left({C}_{t+1} \left(u\right)- {C}_{t} \left(u\right)\right)\le \tau$$

Expression 13 minimizes the contact between individuals with a high difference in contagion potential (CP), by grouping nodes with similar CP. Given the location of node *u* at time *t*, *C*_*t*_(*u*) = (*x*_*t*_ (*u*)*, y*_*t*_ (*u*)), Inequality 14 ensures that distance between the current location of any node *u* at time *t*, *C*_*t*_(*u*), and his location at *t* + 1, *C*_*t*+1_(*u*) is bounded by the distance threshold *τ* feet.

### Scalable solutions

In the optimization strategies (see “Social distancing optimization” section), we are looking for the next location of each individual in the social graph *G*, such that the optimization goals (Eqs. 11 - 14) may be met. Therefore, the optimizer must output vector *C* = [*C*_1_*, C*_2_*,* · · ·*, C*_*n*_], where *C*(*u*) = (*x*(*u*)*, y*(*u*)) is the coordinate of individual *u*. This raises scalability challenges for large **n**. To address this, we propose two scalable social distancing strategies, namely the sampling and grid-based strategy. We define *time epoch* as follows:

- *Sampling* *approach.* similar to MCMC Gibbs Sampling, all nodes at- tempted to re-locate exactly once within the epoch, assuming all other nodes are fixed
- *Grid-based* *approach.* one run of the optimization approaches (discussed in “Social distancing optimization” section) within the grid

For the direct contact, clustering, and contagion potential approaches, we calculate *scores* on social network as Expressions 11, 12 and 13, respectively, and the optimization goals are to minimize these scores calculated in all three optimizations based on the (1) social ties between S and I nodes, (2) triangles with S and I nodes and (3) difference in CP of connected nodes, respectively.

#### Sampling strategy

**Fig. 6:**
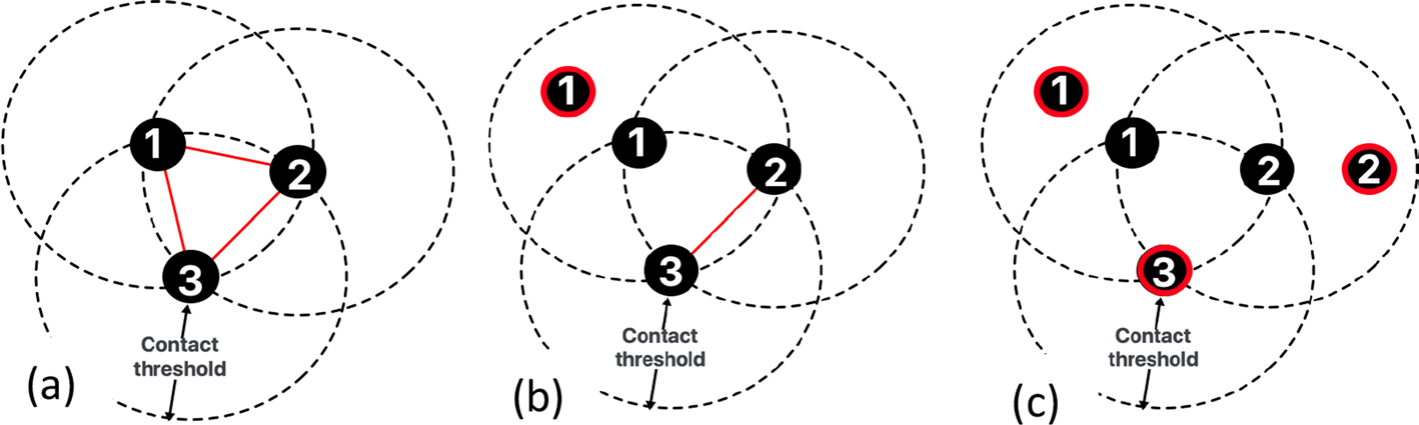
Sampling on 3-node network: **a** original location, **b** move node 1, **c** move node 2 but reject movement of 3

The sampling strategy is inspired by the Markov Chain Monte Carlo (MCMC) approach, namely Hastings-Metropolis (Carlo 2004). In each time epoch t and social network $G\_t,$ we iteratively sample a node u at a time with equal probability and attempt to re-locate it if other nodes V ($G\_t$)\u do not move. The optimizer is invoked to place u at locations within radius τ of current location Ct(u). The move is accepted if the resultant social network minimizes scores and rejected otherwise. The time epoch is complete when the convergence criteria, the fraction of total relocated nodes $VR\left({G}_{t}\right)$ is less than a threshold π, i.e., $\frac{VR\left({G}_{t}\right)}{V\left(G\right)}< \pi$, is satisfied. In Fig. 6, we demonstrate the above steps for a 3-node social network.

#### Grid-based strategy

**Fig. 7:**
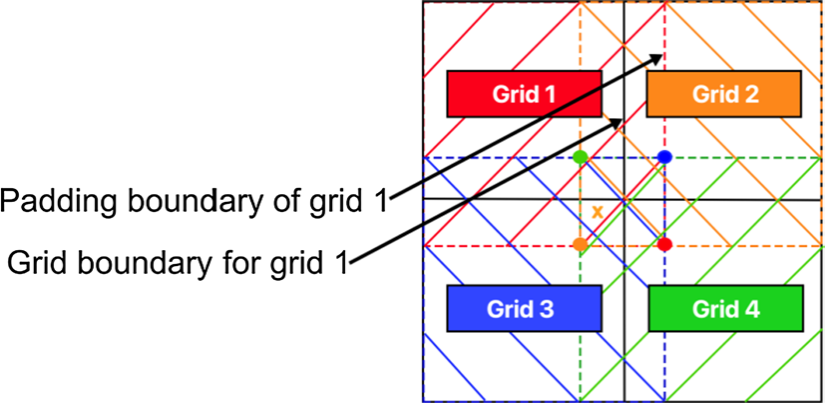
Deployment region partitioned into 4 grids. Each grid has a padding boundary; if the current location of a node (marked by orange cross) is outside the boundary of current grid 2, it is reassigned to grid 3 since its location belongs to the latter’s grid boundary

We parallelize the optimization approaches by partitioning the deployment region into grids. Figure 7 depicts a scenario where the region is partitioned into 4 grids colored red, orange, blue and green. At each time epoch, every grid with the nodes placed within it at the time is initialized in a parallel process. Each grid has a padding region, whose area extends from the horizontal and vertical border of each grid, by length equal to the distance threshold $\tau$. Since any node can move a distance of $\tau$ in each epoch, a node belonging to a grid experiences an illusion that it is not restricted by the grid boundary and can reside anywhere within the padded boundary (see Fig. 7).

##### Approach

We consider a master–slave paradigm running a map reduce approach. Given *Z* spatial grids, in a time epoch, the master *maps* grids to parallel slave processes and the optimization approaches are simultaneously and independently invoked in the different grids. At the end of an optimization run, the processes return the optimized locations from their grids to the master. The master performs the *reduce* step where any node *u*, with location *C*(*u*), located (outside its grid boundary and) in padded region is assigned to another grid if *C*(*u*) belongs to the grid’s boundary. Consider the location of a node, marked in orange cross, originally belonging to grid 2 is reassigned to grid 3 as its new location is within the latter’s grid boundary.

#### Hybrid strategy

We combine the sampling- and grid-based approach in order to achieve greater scalability. We follow three steps: (1) the deployment region is partitioned into grids, (2) within each grid the sampling strategies are invoked by master, and (3) slave processes return the optimized locations of its nodes once the sampling convergence criterion is achieved. The above steps are repeated in each time epoch.

##### Observations

Note that high convergence index or grid count in the sampling and grid-based approaches result in greater speed-up at the expense of optimality of the optimization goal (as shown in the experimental results in “Results” section). In the rest of the paper, we use the term *approach* to refer to the three optimizations (“Social distancing optimization” section) and the term *strategy* to refer to sampling- (or distributed) and grid-based solutions.

## Results

We create a platform on Python SimPy discrete event simulation environment (Matloff 2009), where each node is an agent, and the total time is divided into discrete time epochs. The simulation environment enforces the differential equations of the SEIRD epidemic model (Eqs. 1–4) indirectly as follows: the social network at any time epoch is implemented through a spatial model, where moving agents are nodes that form a temporal social tie when they are within contact threshold $\text {d}$﻿. This section has the following subsections: (1) optimization versus sampling approaches, (2) effect of convergence index, the performance of (3) distributed strategy, and (4) grid-based solution and (5) scalability analysis (Table 1).

**Table 1:** Default parameter values

Parameter	Notation	Value
Number of iterations	-	25
Population size	*ν*	50–4000
Simulation area length	*X*	50–400 ft.,
Simulation area breadth	*Y*	50–400 ft
Simulation duration	*T*	50*,* 100 time epochs
SEIRD parameters (Korolev 2021)	*σ, γ, α, β*	0*.*25*,* 0*.*1*,* 0*.*05*,* 0*.*55
Contact threshold	*d*	6 ft
Distance threshold	*τ*	25 ft
Init. sus. and inf. fraction	-	0*.*7*,* 0*.*3
Convergence index	*π*	0*.*3–0*.*5
Grid count	*Z*	1–64

### Default parameters

We carry out experiments on 2.6 GHz 6-Core Intel Core i7 macOS 16 GB RAM, each of duration 100 time epochs, on a population ranging from 15–4000 individuals and contact rate β = 0.55. We plot mean curve from 25 iterations, showing the cumulative count, which we measure as the sum of infected, recovered, and dead individuals at a given time. To ensure fairness of comparison, individuals have the same initial starting location and epidemic status in each run of the experiment. The contact threshold is ${\text {d}} = 6 \,{\text{ft}}$. and individuals move within distance threshold τ = 25 ft. on an average at every time epoch. All three strategies are run using the SEIRD model for contagion spread. We compare the scalable and distributed solutions against the random mobility strategy. Grid-based parallelization was achieved using the Python Multiprocessing library (Palach 2014). It is worth repeating that we define scores for the scalable versions of the three optimization approaches in terms of the values of expressions 11, 12 and 13; the lower the score, the closer the scalable solution is to the optimal solution (Table 2).

**Table 2:** No. of nodes vs. area

Nodes	75	90	105	120	135	150
Area (sq. ft)	100 × 50	100 × 60	100 × 70	100 × 80	100 × 90	100 × 100

### Optimization versus sampling approaches

**Fig. 8:**
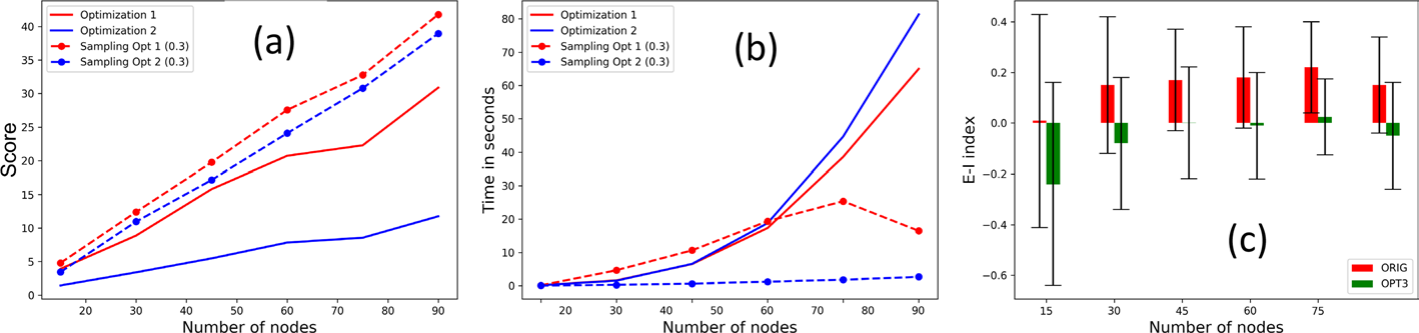
Sampling strategy: Comparison of **a** scores for optimization versus sampling approaches, **b** running time for optimization versus sampling approaches; **c** E-I scores for original and optimized social networks. (Optimizations 1, 2 and 3 refer to direct contact, clustering, and contagion potential approaches, respectively.)

We calculate the running time and scores of direct contact and clustering approaches against the corresponding sampling approaches with convergence index 0.3. Figure 8a shows that sampling strategy for direct contact approach is much closer to its optimization counterpart than clustering approach. With respect to the running time in seconds, the optimization approaches exhibit a significantly higher growth rate than the sampling versions (see Fig. 8b). We apply nonlinear curve-fitting to fit the running time to polynomials of order 2 (i.e., $y = {c}_{0}+ {c}_{1}{x}_{1} + {c}_{2}{x}_{2}$). In Table 3, we show that the direct contact and clustering approaches have higher coefficient or order 2 (${c}_{2}$), resulting in higher running time than the sampling counterparts.

**Table 3:** Polynomial coefficients from the nonlinear curve fitting (of order 2) on the running time in seconds against the number of nodes, for approach 1 (i.e., direct contact) and approach 2 (i.e., clustering)

Approach	Coeff. 0 (*c*_0_)	Coeff. 1 (*c*_1_)	Coeff. 2 (*c*_2_)
Opt-1 (Direct contact)	0*.*98	− 4*.*24	3*.*39
Sam-1 (Direct contact)	− 2*.*62	11*.*70	− 1*.*59
Opt-2 (Clustering)	1*.*82	− 7*.*54	4*.*63
Sam-2 (Clustering)	0*.*04	0*.*08	0*.*07

Recall from “Key Network Science Concepts” section, homophily of a network is measured in terms of E-I index. Since optimization contagion potential approach (Expression 13) attempts to achieve homophily by grouping nodes with similar CPs. We compare the E-I indices of the original and (sampling approach) modified networks. We discretize the node CPs by rounding them off to one decimal place (i.e., 0.05 becomes 0.1) and record the E-I indices. Figure 8c shows that the E-I indices of the optimized networks are significantly lower (even negative), suggesting that they exhibit a higher proportion of links among nodes with similar CPs.

### Effect of Convergence Index

**Fig. 9:**
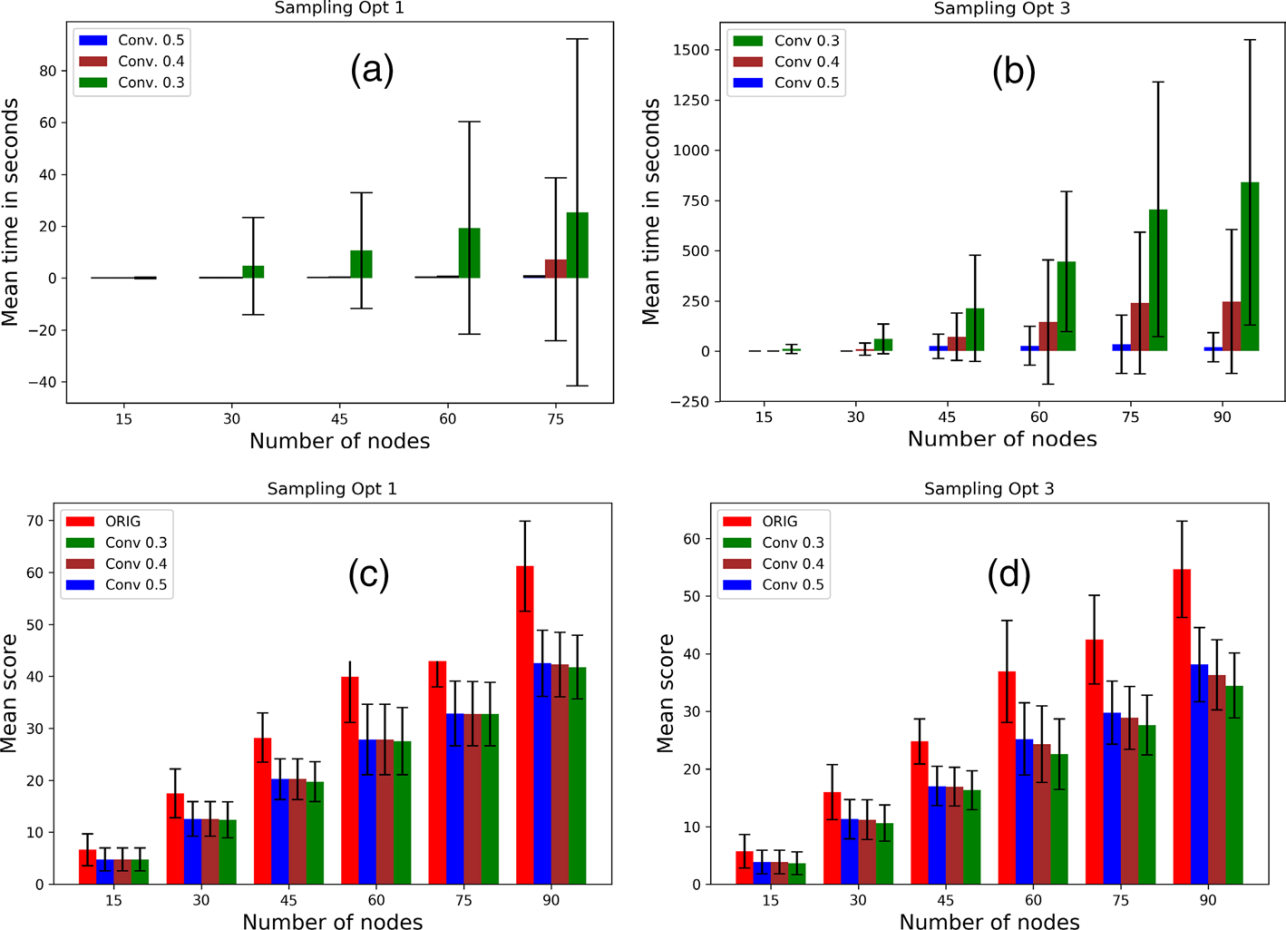
Effect of convergence index: For varying convergence indices 0*.*3*,* 0*.*4*,* 0*.*5, mean scores for sampling **a** direct contact approach and **b** contagion potential approach; running time in seconds for sampling **c** direct contact approach and **d** contagion potential approach

We vary the convergence indices—0.3, 0.4, 0.5—and record the running scores and running time for sampling approaches 1 and 3. We study the trade-off offered by the convergence parameter. Figure 9a, b show that starting with the original scores (colored red), the sampling approaches exhibit better scores. For both approaches, lowering in convergence does not cause a major improvement. Figure 9c, d show that, for both approaches, convergence indices 0.4 and 0.5 greatly outperform 0.3 in terms of running time.

### Performance of distributed strategy

**Fig. 10:**
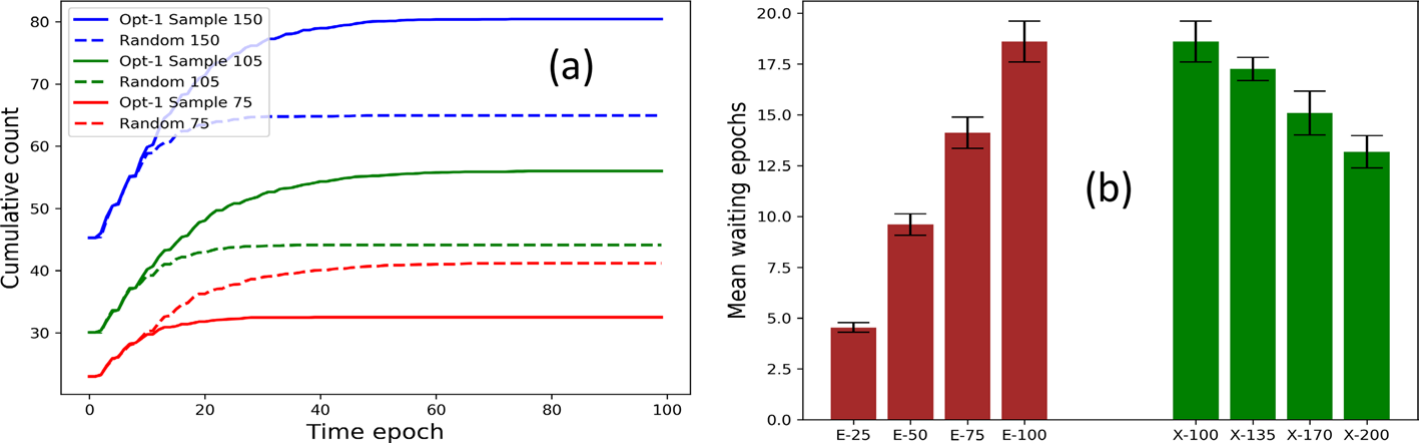
Distributed strategy: Comparison of **a** cumulative counts (comprising infected, recovered, and dead individuals) of distributed approach against random mobility **b** running time for optimization versus sampling approaches. (Optimizations 1, 2 and 3 refer to direct contact, clustering, and contagion potential approaches, respectively.)

We estimate the performance of the distributed approach (defined in “Scalable solutions” section) with respect to the cumulative count (constituting infected, recovered, and dead population) and waiting times needed to wait for their neighbors with lower IDs to move. Figure 10a shows that for 75, 105, 100 nodes, the distributed approach exhibits a lower cumulative count (i.e., slower contagion) over time.

We also consider the two variables that may result in variable waiting times – number of nodes and deployment area. Figure 10b shows that (1) varying population 25, 50, 75, 100 for fixed area 100 × 100 sq. ft. causes a linear growth in mean waiting epochs per node (brown bars); similarly, (2) for a fixed population of 100 individuals and varying the area of 100 × 100, 135 × 100, 170 × 100 and 200 × 100 results in a decrease in the mean waiting time epochs per node.

**Fig. 11:**
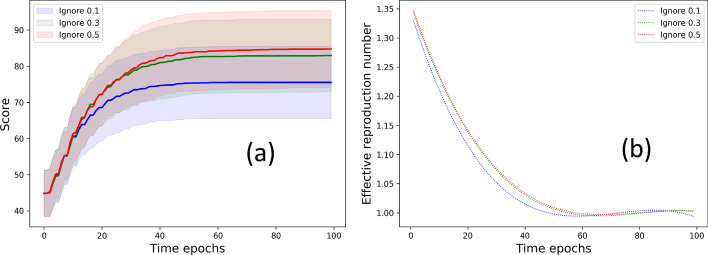
Flouting recommendations: **a** Score for *direct contact approach* and **b** effective reproduction number (smoothed by the Savitzky–Golay filter) for variation in fraction of times nodes flout the optimizer recommendation

*Flouting recommendation.* We study the effect of flouting the location recommendation of the social distancing strategy. We record the score when the nodes follow sampling approach 1 for 90%, 70%, 30% of time and thereby undertake random mobility 10%, 30%, 50% at other times. Figure 11a shows that scores are hampered as the nodes increasingly ignore recommendation. This result may also be viewed in light of situations where certain nodes may temporarily get discharged or fall off the grid and lose contact with their immediate neighbors. Furthermore, we plot the effective reproduction number (refer to “SEIRD Epidemic Model” section for details) that provides a realistic measure of the number of secondary cases caused per infected individual. Figure 11b shows that the effective reproduction number (smoothed by the Savitzky–Golay filter (Press and Teukolsky 1990)) is the least when the population obeys the mobility recommendations.

### Performance of grid-based solution

**Fig. 12:**
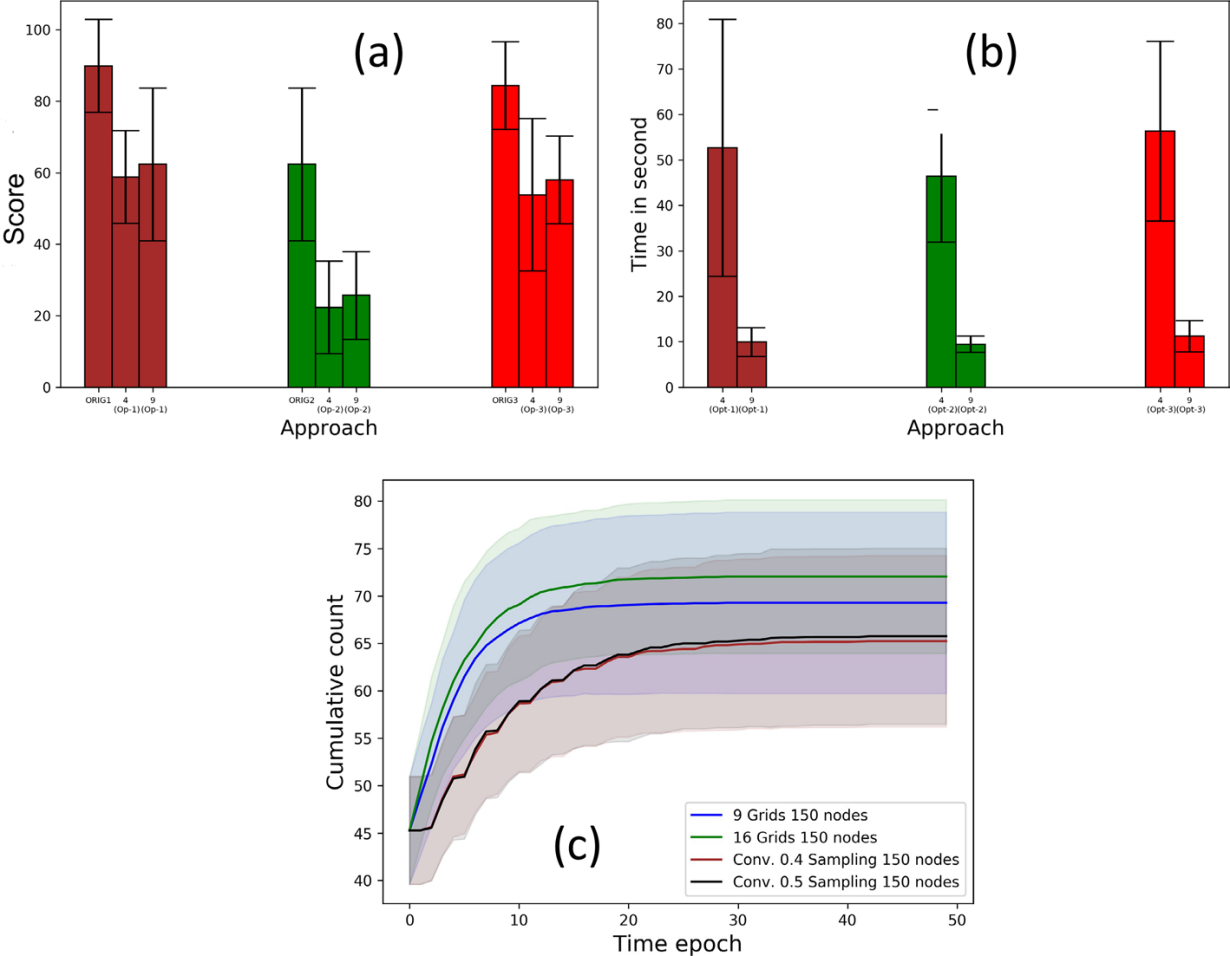
Grid based solution: For approaches 1, 2 and 3 with 4 and 9 grids **a** optimization score, **b** running time in seconds; **c** comparison of cumulative count for grid strategy (9*,* 16 grids) and sampling approach (convergence index 0*.*4*,* 0*.*5)

The goal of the grid-based solution is to achieve a running time vs. performance trade-off. We evaluate the efficacy of the grid-based approach with respect to the score and running time in seconds for varying grid counts. For a social network of 150 nodes, Fig. 12a shows the mean original score and the improved scores with 4 and 9 grids with direct contact approach; the scores achieved by the two grid configurations are comparable. With respect to the running time in seconds, Fig. 12b shows that the 9-grid configuration yields approximately 5 times speed up compared to the 4-grid counterpart, proving the efficacy of the parallel solution.

We compare the performance of the sampling and grid-based solutions with respect to the cumulative counts. Recall from our discussion in “Sampling strategy and Grid-based strategy” section that ﻿a (1) high convergence index in the sampling approach or (2) large number of grids in the grid-based approaches, yield higher speed-up at the cost of the optimality of the optimization objectives. Figure 12c shows that the sampling strategy with convergence indices 0.4, 0.5 achieve a significantly lower mean count than 4- and 9-grid configurations for networks of 25 nodes.

### Scalability analysis

**Fig. 13:**
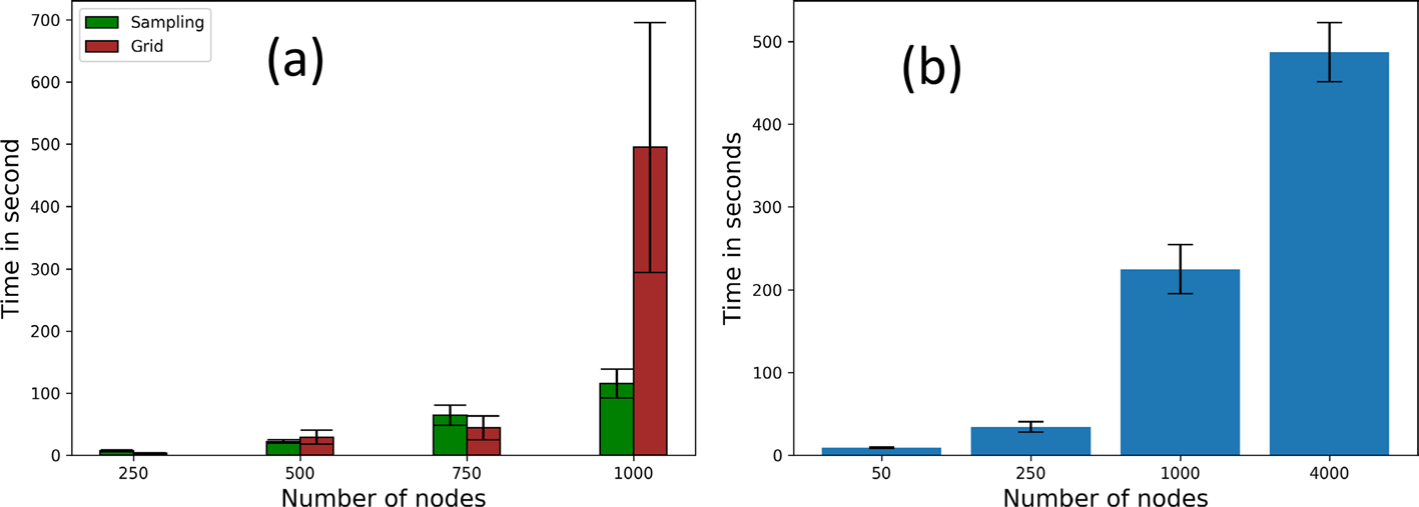
Scalability on direct contact approach: time in seconds for **a** grid strategy (16 grids) and sampling strategy (conv. 0.4), **b** hybrid approach on 50, 250, 1000, 4000 nodes with 1, 4, 16, 64 grids

We analyze the improvement achieved in running time due to the grid and sampling approaches by recording the running time for 250, 500, 750, 1000 nodes with area 200 × (1) 50, (2) 100, (3) 150 and (4) 200 sq. ft., respectively. For the sampling and grid strategies we use convergence index 0.4 and 16 grids, respectively. Figure 13a shows that, for direct contact approach, the sampling strategy scales better than the grid-based strategy.

We also evaluate how the running time compares for a hybrid of sampling and grid strategies on direct contact approach, particularly when more computational resources are employed. Recall from “Hybrid strategy” section, the hybrid approach uses grid-based parallelization and the sampling approach with the grids. We consider four settings (summarized in Table 4). Figure 13b shows that the running time for the hybrid strategy grows proportionally with the order of the social network. The growth rate in this experiment, with increasing computational resources, is lower than that reported in Fig. 13a where the number of grids is kept constant.

**Table 4:** No. of nodes vs. area

Nodes	50	250	1000	4000
Area (sq. ft)	50 × 50	100 × 100	200 × 200	400 × 400
Grids	1	4	16	64

## Discussions

Our simulations suggest that the proposed strategies mitigate the scalability challenges of solving the three optimizations for large populations. In addition to the speed up exhibited by the sampling, grid-based and hybrid strategies, the distributed algorithm enables each node to operate solely on the knowledge of immediate neighbors, as opposed to the entire social network topology. It however raises a few questions and offers new research directions. First, the dynamics of mobility in an urban setting is highly noisy (characterized by (1) erratic movements and (2) uneven spatial population density), making it imperative to deploy the system in a real setting to study their running time complexity and load balancing. To achieve this, we have designed a MyCovid mobile application (Roy et al. 2021d; Roy 2021) that is currently being used by a small population of students to validate the original optimization strategies. Second, although we have tested the optimization on human mobility models, we will need to incorporate the fact that individuals may have predetermined source and destination locations that may override the recommendations of the optimization strategies. This requires an online algorithm to learn personalized schedules and itineraries to make informed recommendations. Similarly, we shall compare the performance of the proposed strategies for small and large population sizes. For accurate predictions with small population, we shall incorporate the necessary correction factors (Grima 2010). Third, there are important security and privacy considerations associated with location sharing. Although the distributed strategy annuls the need to know the entire social network topology, individuals may exhibit reluctance to be detected by neighbor devices, making it essential to build adaptive models that can work with uncertainty as well as infuse identity detection and privacy-preserving techniques into the system. Fourth, it is worth exploring dynamic algorithms that autonomously adjust system parameters like convergence index and grid count (or size) based on the influx or outflow of nodes in urban space.

## Conclusion

﻿In this paper, we presented scalable and distributed social distancing strategies to inform the mobility of individuals roaming in an urban space. The proposed strategies leverage network science principles, such as homophily and network clustering, in conjunction with MCMC Gibbs random sampling and grid-based spatial parallelization. In addition to scaling well for large social networks, the distributed strategy allows individuals to determine next locations without knowledge of the entire network topology. We perform simulation experiments to delineate how one can tune system parameters such as convergence index and grid count to achieve trade-off between running time and rate of contagion. We compare the performance of the proposed strategies, as well as their hybrid, against random human mobility for varying human population sizes and analyze how ignoring optimization recommendations affect overall infection spread.

## Data Availability

All relevant data (epidemiological and demographic data related to the boroughs of New York City) as well as the Python scripts are made available at https://github.com/satunr/COVID-19/tree/master/Network%20Science/ ScriptScalable.

## Abbreviations

LSTM: Long Short-Term Memory
S: Susceptible
E: Exposed
I: Infected
R: Recovered
D: Death
CP: Contagion potential
MCMC: Markov Chain Monte Carlo
